# Characterization of the 3,4-Dichloroaniline Degradation Gene Cluster in *Acinetobacter soli* GFJ2

**DOI:** 10.3390/microorganisms12030613

**Published:** 2024-03-19

**Authors:** Namiko Gibu, Daisuke Kasai, Saki Sato, Michiro Tabata, Alisa Vangnai, Masao Fukuda

**Affiliations:** 1 Department of Materials Science and Bioengineering, Nagaoka University of Technology, Nagaoka 940-2188, Niigata, Japan; 2 Department of Biochemistry, Faculty of Science, Chulalongkorn University, Bangkok 10330, Thailand

**Keywords:** *Acinetobacter soli*, 3,4-dichloroaniline degradation, phenylurea herbicide bioremediation

## Abstract

3,4-Dichloroaniline (34DCA), a major metabolite of phenylurea herbicides, causes environmental contamination owing to its toxicity and recalcitrant properties. *Acinetobacter soli* strain GFJ2, isolated from soil potentially contaminated with herbicides, can degrade 34DCA. This study aimed to identify and characterize the 34DCA degradation gene cluster responsible for the conversion of 34DCA to 4,5-dichlorocatechol in the strain GFJ2. Genome analysis revealed one chromosome and seven plasmids in GFJ2, comprising 21, 75, and 3309 copies of rRNA, 75 tRNA, and protein-encoding genes, respectively. A gene cluster responsible for 34DCA degradation was identified, comprising *dcdA*, *dcdB*, and *dcdC*, which encode dioxygenase, flavin reductase, and aldehyde dehydrogenase, respectively. Transcriptional analysis indicated that this gene cluster is constructed as an operon, induced during 34DCA utilization. The heterologous expression of *dcdA* and *dcdB* in *Escherichia coli* confirmed their activity in degrading 34DCA to an intermediate metabolite, converted to 4,5-dichlorocatechol via a reaction involving the *dcdC* gene product, suggesting their involvement in 34DCA conversion to 4,5-dichlorocatechol. Deletion mutants of *dcdA* and *dcdB* lost 34DCA degradation ability, confirming their importance in 34DCA utilization in GFJ2. This study provides insights into the genetic mechanisms of 34DCA degradation by GFJ2, with potential applications in the bioremediation of environments contaminated by phenylurea herbicides.

## 1. Introduction

Phenylurea herbicides, including diuron and linuron, are commonly used as herbicides or pesticides and contain a single chlorinated phenyl ring with a substituted urea moiety. They are widely used to protect agricultural plants, including cotton, fruits, cereals, field crops, and orchards. Diuron is generally found at trace concentrations of milligrams per liter in drainage water from agricultural soils [1]. It has been included in the European Commission’s list of priority substances for European freshwater resources (European Commission, 2000) and the U.S. Environmental Protection Agency’s Second Drinking Water Contaminant Candidate List (U.S. Environmental Agency, 2005). In addition to diuron, the main metabolite, 3,4-dichloroaniline (34DCA), has been detected in natural waters [2,3], posing 34DCA environmental concerns owing to its toxicity and recalcitrant properties [4]. Therefore, the characterization of mono- and dichloroaniline biodegradation is important for the remediation of environments contaminated by phenylurea herbicides.

Some bacteria capable of degrading diuron and linuron to mono- or dichloroaniline, such as 34DCA and 4-chloroaniline, have been reported [5,6,7]. *Sphingomonas* sp. strain Y57, isolated from the wastewater treatment system of an herbicide factory, degrades 34DCA to 4,5-dichlorocatechol via a hydroxylation reaction [8]. *Variovorax* sp. SRS16 and WDL1, isolated from a bacterial consortium obtained after enrichment in Danish agricultural soil, degrades linuron via 34DCA [9,10]. Notably, proteomic and transcriptional analyses have revealed that amidase and multicomponent dioxygenase genes are involved in linuron degradation in strain SRS16 [11]. Nitisakulkan et al. reported that the toluene dioxygenase complex (TodC1C2BA) from *Pseudomonas putida* T57 is involved in the degradation of 4-chloroaniline to 4-chlorocatechol and 2-amino-5-chlorophenol. The introduction of the *tod* operon (including *todC1C2BADE*) enhanced 4-chloroaniline degradation, suggesting that *tod* genes are required for 4-chloroaniline degradation in strain T57 [12]. Furthermore, it has been reported that *Bacillus megaterium* IMT21 degrades 34DCA via an intermediate acetanilide metabolite [13]. The pathways and related enzymes of aniline degradation have been reported in various bacteria [14].

*Acinetobacter soli* GFJ2 (formerly described as *A. baylyi* BCC 56596 or NBRC 109690) can degrade various halogenated anilines, and has been isolated as a 4-chloroaniline utilizing bacterium from soil from fruit peels that were potentially contaminated with herbicides [15]. This strain can degrade a wide range of chloroanilines. Therefore, strain GFJ2 has potential applications in the bioremediation of environments contaminated with phenylurea herbicides. It has been reported that this strain degrades 34DCA to 4-chloroaniline via a dechlorination reaction. The resulting 4-chloroaniline is thought to be degraded to aniline or 4-chrolocatechol, which are further degraded via the catechol/chlorocatechol 1,2-cleavage pathway [15]. However, the genes involved in the degradation of 34DCA to chlorocatechol have not yet been identified. Therefore, the present study aimed to identify and characterize the 34DCA degradation gene cluster containing the *dcd* genes of the strain GFJ2, responsible for the conversion of 34DCA to 4,5-dichlorocatechol. 

## 2. Materials and Methods

### 2.1. Bacterial Strains, Culture Conditions, and DNA Manipulations

*A. soli* GFJ2 (BCC 56596 or NBRC 109690) and its mutant derivatives were grown at 30 °C in Luria–Bertani (LB) medium or W minimal salt medium [16] containing 10 mM succinate with or without 1 mM 34DCA. *Escherichia coli* strains were grown in LB medium at 37 °C. For the culture of cells carrying antibiotic resistance markers, the media were supplemented with ampicillin (100 mg/L) or kanamycin (25 mg/L).

DNA manipulations, including genomic DNA isolation, nucleotide sequencing, and nucleotide sequence analysis, were performed as previously described [17,18].

### 2.2. Genome Sequence Analysis

After the extraction of genomic DNA from strain GFJ2, DNA quality and quantity were assayed using a Qubit 2.0 fluorometer (Life Technologies, Carlsbad, CA, USA). Genomic DNA was sequenced using single-end sequencing with a 454 GS FLX Titanium system (Roche, Basel, Switzerland), paired-end sequencing with a MiSeq system (Illumina, San Diego, CA, USA), and a PacBio RS II sequencer (PacBio, Menlo Park, CA, USA). Sequencing data were assembled using Newbler ver. 2.6 (Roche). Annotation was performed using the NCBI Prokaryotic Genome Annotation Pipeline ver. 3.1 [19] and the RAST server [20]. The rRNA and tRNA genes were predicted using RNAmmer software ver. 1.1 [21] and tRNAscan-SE Online [22], respectively. Homology searches were carried out using the DDBJ database with the BLAST program (https://blast.ncbi.nlm.nih.gov/Blast.cgi, 25 January 2020). Pairwise alignment was performed with the EMBOSS alignment tool (http://www.ebi.ac.uk/emboss/align/, 30 March 2020).

### 2.3. Construction of Disruption Mutants

*dcdA* and *dcdB* were disrupted using the *sacB* counterselection system, as previously described [23,24]. The N- and C-terminal portions of *dcdA* or *dcdB* were inserted in tandem into pK18*mobsacB*. Each of the resulting plasmids was introduced into GFJ2 to allow integration into the wild-type gene locus on the chromosome by homologous recombination. The corresponding deletion mutant was selected as a sucrose-tolerant derivative generated by additional homologous recombination that eliminated part of the target gene and the vector sequence containing *sacB*. Disruption of these genes was confirmed by performing diagnostic PCR or Southern hybridization analysis.

### 2.4. Reverse Transcription PCR and Quantitative Reverse Transcription PCR

The cells of strain GFJ2 were grown at 30 °C in 100 mL of W medium containing 10 mM succinate, with or without 1 mM 34DCA. After the incubation, the resulting cells were harvested using centrifugation, washed with RNA protect bacterial reagent (QIAGEN, Hilden, Germany), and stored at −80 °C. Total RNA was extracted from the cells as previously described [25]. Purified RNA was incubated with 1 U of RNase-free DNase I (Takara Bio, Kusatsu, Japan) to remove the contaminating genomic DNA from the samples.

Single-stranded cDNA was synthesized from 1.0 µg RNA using ReverTra Ace reverse transcriptase (Toyobo, Osaka, Japan) with random hexamer primers in a 30 μL reaction mixture. The cDNA was subjected to reverse transcription (RT)-PCR and quantitative (q)RT-PCR analyses. A qRT-PCR analysis was carried out using dcdA_F (ACTGACGGCCGAGTATGAGC)- and dcdA_R (GCACGCTGTGGCTTATCGAA)-specific primers with a StepOne real-time PCR system (Thermo Fisher Scientific, Waltham, MA, USA). To normalize the amount of RNA in each sample, the 16S rRNA gene was used as an internal standard, as previously described [26].

### 2.5. Expression of dcd Genes in E. coli

The coding regions containing *dcdA*, *dcdA*-*dcdB*, and *dcdC-dcdA-dcdB* were independently amplified using PCR. The amplified fragments were separately cloned into pUC19. The resulting plasmids were independently introduced into *E. coli* DH5α cells and the transformants were grown at 37 °C in an LB medium containing ampicillin. The absorbance of the culture was measured using a Biospectrometer (Eppendorf, Hamburg, Germany). When the absorbance of the culture at 600 nm (*A*_600_) reached 0.5, 1 mM isopropyl-β-D-thiogalactopyranoside was added, and the cultures were further incubated for 16 h. The cells were harvested by centrifugation and resuspended in 50 mM Tris-HCl (pH 7.4). The resting cells were used for the 34DCA degradation assays.

### 2.6. Determination of Substrate Residue (34DCA)

To determine the function of the *dcd* gene products, resting *E. coli* cells harboring each *dcd* gene and GFJ2 and their deletion mutants (OD_600_ = 4.0) were incubated with 100 μM 34DCA in a 5 mL assay mixture containing 50 mM Tris-HCl (pH 7.4). The reaction mixture was incubated at 30 °C. After incubation, the concentration of the remaining substrate was measured using high-performance liquid chromatography (HPLC) analysis (1260 Infinity LC system, Agilent Technologies, Santa Clara, CA, USA) with a TSKgel ODS-80 column (6 by 150 mm; Tosoh, Tokyo, Japan). After filtration, 5.0 μL of the reaction mixture was applied. A mixture of water (60%) and acetonitrile (40%) (both containing 0.1% formic acid) was used as the mobile phase for HPLC analysis, at a flow rate of 1 mL/min. 34DCA, compound I, and 4,5-dichlorocatechol were detected at 240 nm, and their retention times were 2.24, 2.34, and 2.08 min, respectively. The concentrations of 34DCA and 4,5-dichlorocatechol were determined from a calibration curve created from the area of the standard substance at the specified concentrations. The amount of compound I was calculated as a relative value, using the area at the time of greatest accumulation as 100%.

### 2.7. Accession Numbers

The genome sequence of *A. soli* strain GFJ2 was deposited in DDBJ/EMBL/GenBank under the accession numbers CP016896, CP016897, CP016898, CP016899, CP016900, CP016901, CP016902, and CP016903 for chromosomes pGFJ1, pGFJ2, pGFJ3, pGFJ4, pGFJ5, pGFJ6, and pGFJ7, respectively.

### 2.8. Statistical Analysis

All assays were repeated three times. Data are presented as the means ± the standard deviations (SDs) of independent experiments. Error bars represent standard error. The levels of significance were calculated using independent Student’s *t*-tests, and the results were considered statistically significant at *p* < 0.05.

## 3. Results

### 3.1. Determination of the Genome Sequence of Strain GFJ2

To understand the genetic background of GFJ2, its genome sequence was determined using MiSeq, a PacBio RS II system, and Sanger sequencing. The final complete sequence of the GFJ2 genome consisted of one chromosome (3,438,298 bp; 43.1% G+C) and seven plasmids (pGFJ1: 86,373 bp, 38.9% G+C; pGFJ2: 78,649 bp, 37.0% G+C; pGFJ3: 10,453 bp, 37.0% G+C; pGFJ4: 9416 bp, 38.7% G+C; pGFJ5: 5086 bp, 39.4% G+C; pGFJ6: 4066 bp, 38.2%G+C; and pGFJ7: 4008 bp, 37.3% G+C). The genome contained 21 rRNAs, 75 tRNAs, and 3309 protein-encoding genes. These sequences were deposited in GenBank under accession numbers CP016896.1 to CP016903.1.

### 3.2. Identification of 34DCA-Degrading Genes

**Figure 1:**
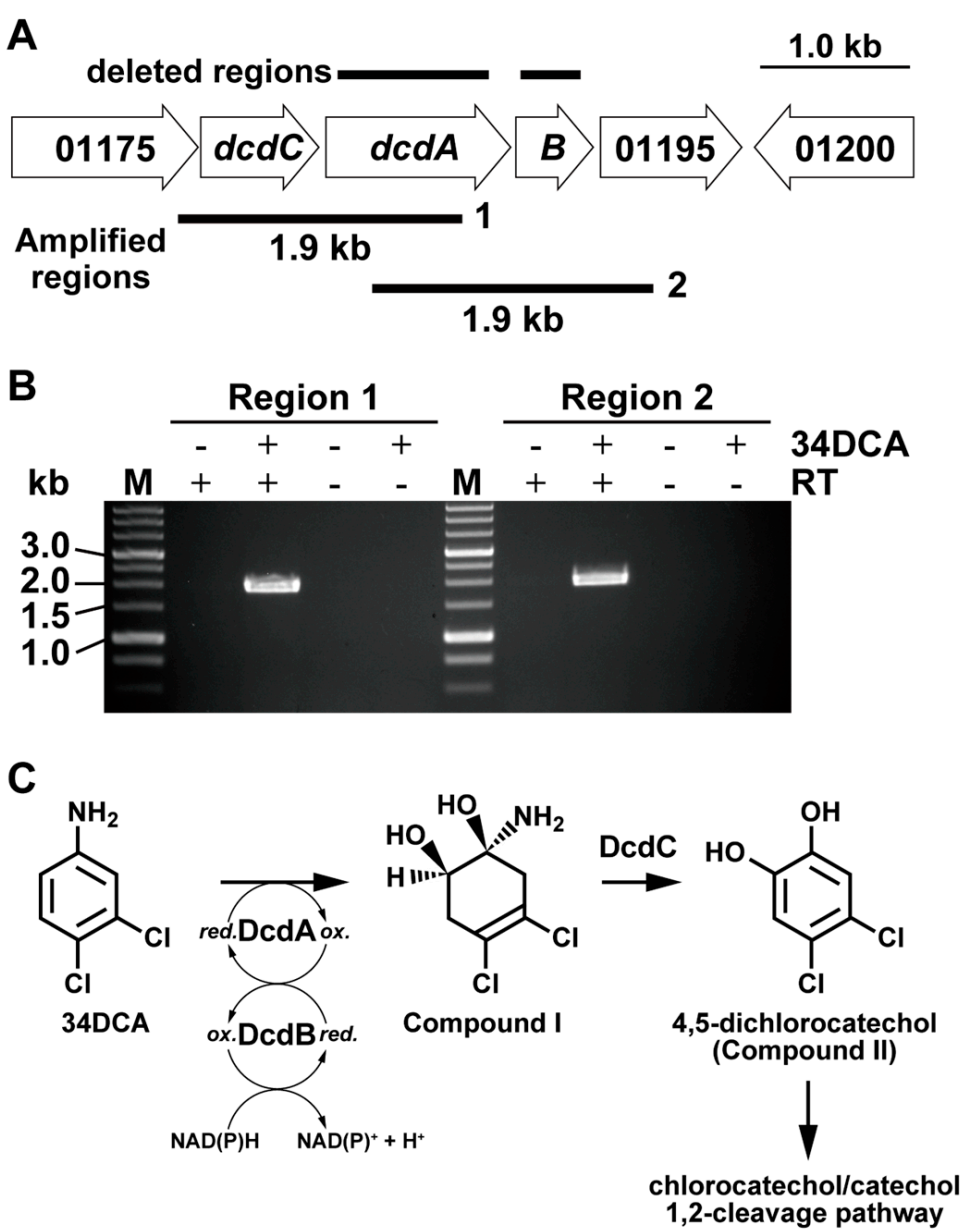
Organization of the *dcd* gene cluster, and a putative 34DCA catabolic pathway in GFJ2. (**A**) Organization of the *dcd* genes. Open arrows indicate the sizes, locations, and transcriptional directions of the open reading frames. Boldfaced bars below the gene cluster diagram represent the locations of the amplified RT-PCR products shown in panel c. Boldfaced bars above the *dcd* genes indicate the regions deleted in the *dcd* gene mutants. (**B**) RT-PCR analysis of the *dcd* gene cluster in GFJ2. The results of agarose gel electrophoresis of RT-PCR products obtained with primers targeting regions 1 and 2 are shown. Positions of primer pairs are indicated. Lane M, molecular weight markers; lanes + and -, RT-PCR with and without RT, respectively. (**C**) The proposed catabolic pathway of 34DCA in GFJ2.

Two pathways have been proposed for the degradation of 34DCA. The first is the pathway in which 34DCA is converted by dechlorination to 4-chloroaniline, which is then metabolized by an aromatic ring cleavage reaction. The other is a pathway in which 34DCA is converted to aniline by two dechlorination steps followed by aromatic ring cleavage and then metabolized [15]. The dehalogenase gene that catalyzes the dechlorination of 34DCA was not identified in the NCBI Prokaryotic Genome Annotation Pipeline. Instead, a cluster that contained genes encoding a putative oxygenase (BEN76_01185) and putative flavin reductase (BEN76_01190) was identified on the chromosome. The BEN76_01185 and BEN76_01190 genes were named *dcdA* and *dcdB*, respectively (Figure 1A and Table 1). The elucidated amino acid sequences of *dcdA* and *dcdB* had overall similarities of 87% with IifC and 90% with IifD, respectively, which encode an indole hydroxylase oxygenase and reductase from *Acinetobacter* sp. strain O153, respectively [27]. The identified amino acid sequence of BEN76_RS01180 (*dcdC*), located upstream of *dcdA*, was similar to that of a short-chain dehydrogenase (IifB) in strain O153, suggesting that this gene is involved in the dehydrogenation reaction. Based on their amino acid sequence similarities, these genes may be involved in the dioxygenation of 34DCA and subsequent dehydrogenation of the diol compound to dichlorocatechol (Figure 1C). The amino acid sequences of BEN76_RS01175 and BEN76_RS01195, located near the *dcd* genes, were similar to those of the dienelactone hydrolase family protein (IifA) and phenol degradation protein (IifE), respectively.

### 3.3. Transcriptional Induction of the dcd Gene Cluster

To examine the operon structure of the *dcd* cluster, RT-PCR analysis was performed using total RNA prepared from GFJ2 cells grown on 100 μM 34DCA and 10 mM succinate. Amplification products with the expected sizes of the BEN76_01175-*dcdA* and *dcdA*-BEN76_01195 segments were detected (Figure 1B). This result indicates that the *dcd* genes are included in the same transcriptional unit. No amplification products were detected when RNA isolated from cells grown on succinate was used.

**Figure 2:**
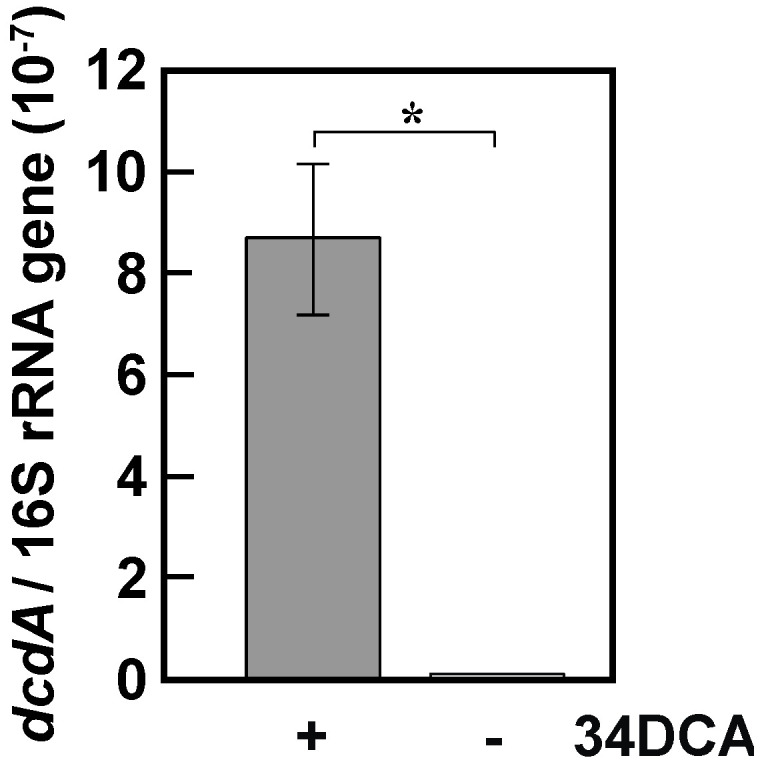
Quantification of the *dcdA* expression levels. Total RNA was isolated from GFJ2 cells grown in W medium containing 10 mM succinate with or without 100 μM 34DCA. The mRNA expression levels were calculated as the ratio of 16S rRNA gene expression. The data are mean values ± standard deviations for four independent experiments. Statistical analysis was performed using Student’s *t*-test. *, *p* < 0.005.

To investigate the transcriptional induction of the *dcd* operon, qRT-PCR was performed using a primer set designed to amplify the internal region of *dcdA*. Total RNA was extracted from GFJ2 cells grown on succinate with or without 34DCA. The levels of *dcdA* mRNA were significantly higher in cells grown in the presence of 34DCA than in cells grown without 34DCA (*p* < 0.05, Student’s *t*-test; Figure 2). This result suggests that the transcription of the *dcd* operon is induced during growth on 34DCA and that this operon is involved in 34DCA degradation.

### 3.4. Disruption of dcdA and dcdB in GFJ2

**Figure 3:**
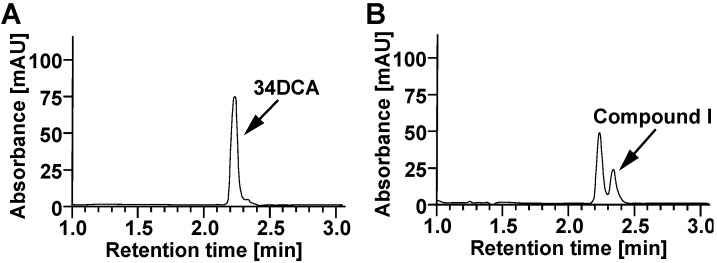
Degradation of 34DCA by resting cells of GFJ2. A reaction mixture containing 100 µM 34DCA and the resting cells of GFJ2 (OD_600_ = 4.0) was incubated at 30 °C. HPLC chromatograms of the reaction mixture at the start and after 6 h of incubation are shown in panels (**A**,**B**), respectively.

**Figure 4:**
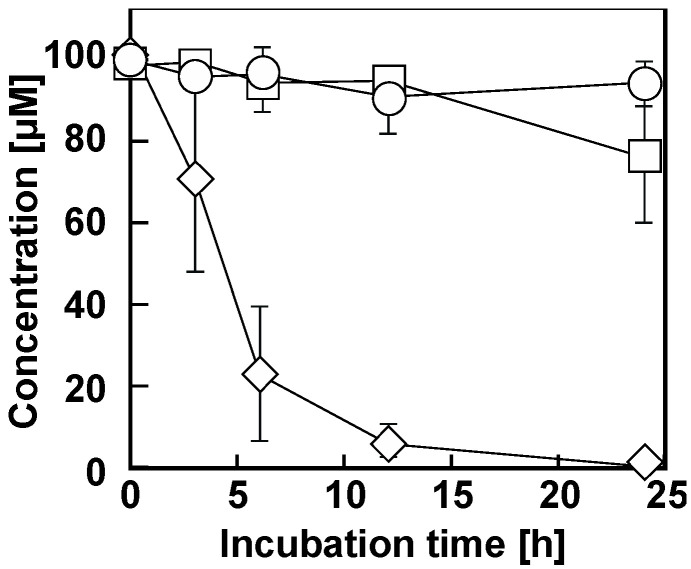
Degradation of 34DCA by resting cells of GFJ2 (diamonds), *dcdA* deletion mutant strain DDA2 (circles), and *dcdB* deletion mutant strain DDB2 (squares). A reaction mixture containing 100 μM 34DCA and resting cells (OD_600_ = 4.0) of each strain was incubated. The remaining amount of 34DCA was determined by conducting HPLC analysis. Each value is the average ± standard deviation of three independent experiments performed in parallel.

To clarify whether *dcdA* and *dcdB* are involved in 34DCA degradation in GFJ2, deletion mutants of *dcdA* or *dcdB* were constructed using gene replacement and disruption plasmids. Resting GFJ2 cells and the deletion mutants were incubated with 34DCA. When the reaction mixture was analyzed using HPLC, the 34DCA peak was detected at a retention time of 2.24 min (Figure 3A). When the GFJ2 cells were incubated with 34DCA, the 34DCA levels significantly decreased after 6 h of incubation (Figure 4). In the same reaction mixture, a peak with a retention time of 2.35 min (compound I) appeared (Figure 3B). This peak disappeared after 24 h of incubation, suggesting that compound I may be an intermediate metabolite of 34DCA. In contrast, the *dcdA*-deleted mutant strain (strain DDA2) and the *dcdB*-deleted mutant strain (strain DDB2) lost the ability to degrade 34DCA (Figure 4). This result indicates that *dcdA* and *dcdB* are essential for 34DCA degradation in GFJ2.

### 3.5. Determination of the Roles of the dcdA, dcdB, and dcdC Genes in 34DCA Degradation

**Figure 5:**
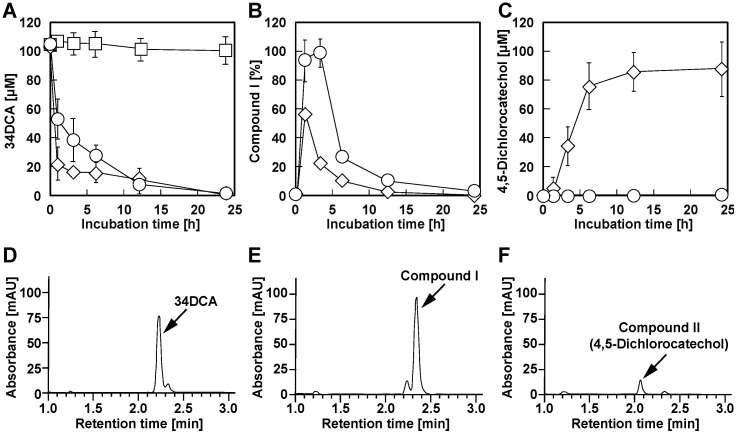
Degradation of 34DCA by the resting cells of *E. coli* expressing *dcdA* (squares), *dcdA-dcdB* (circles), and *dcdA-dcdB-dcdC* (diamonds). (**A**–**C**) 100 μM 34DCA along with the resting cells of *E. coli* containing the *dcd* genes (OD_600_ = 4.0) were incubated. The concentration of 34DCA and compounds I and II (4,5-dichlorocatechol) were determined by performing HPLC analysis. The values represent the averages ± standard deviations of three independent experiments. (**D**–**F**) HPLC chromatograms of the reaction mixture of the resting cells of *E. coli* expressing *dcdA*, *dcdB*, and *dcdC* at the start and after 1 and 12 h of incubation are shown in panels d, e, and f, respectively. The concentrations of 34DCA and 4,5-dichlorocatechol were determined from a calibration curve created from the area of the standard substance at the specified concentrations. The amount of compound I was calculated as a relative value, using the area at the time of greatest accumulation as 100%.

To determine the functions of the *dcdA* and *dcdB* gene products, the resting *dcdA-* or *dcdA*-*dcdB* plasmid-carrying *E. coli* cells were incubated with 100 µM 34DCA, and the reaction mixtures were analyzed using HPLC. No conversion of 34DCA was observed in the reaction mixture expressing *dcdA* (Figure 5A). In contrast, 34DCA levels remarkably decreased after 3 h of incubation with *E. coli* cells expressing both *dcdA* and *dcdB* (Figure 5A). Compound I was also generated in the same reaction mixture (Figure 5B). Compound I disappeared after 24 h (Figure 5B), suggesting that it is an unstable intermediate metabolite generated from 34DCA. These results indicate that the gene products of *dcdA* and *dcdB* are directly involved in the conversion of 34DCA to compound I. 

To characterize the *dcdC* product, the gene was expressed with *dcdA* and *dcdB* in *E. coli.* The resting *E. coli* cells carrying *dcdA*, *dcdB*, and *dcdC* significantly degraded 34DCA after 1 h of incubation (Figure 5A,D,E). The production of compound I (retention time of 2.35 min) was observed in the same reaction mixture (Figure 5B,E). After an additional 12 h of reaction, compound I almost completely disappeared (Figure 5B,F). The same reaction mixture exhibited a peak with a retention time of 2.08 min (compound II; Figure 5C,F). Compound II was not produced in the reaction mixtures containing *dcdA* and *dcdB* (Figure 5C). This result suggests that compound II is generated by the reaction of the *dcdC* gene product (DcdC). When authentic 4,5-dichlorocatechol was analyzed using HPLC, a peak was observed at the same retention time as that of compound II (Supplementary Figure S1). This result strongly suggests that compound II is 4,5-dichlorocatechol, which is thought to be generated from a diol compound (compound I) by DcdC. The peak with a retention time of 1.23 min was also observed even in the absence of substrate, and is, therefore, considered to be a compound derived from the cells.

## 4. Discussion

The present study characterized the functions of genes and enzymes involved in the degradation of 34DCA by *A. soli* GFJ2. To understand the genetic background of this strain, its genome was sequenced, revealing the *dcd* genes responsible for 34DCA degradation. The deduced amino acid sequences of *dcdA*, *dcdB*, and *dcdC* were similar to those of IifC, IifD, and IifB, respectively, of *Acinetobacter* sp. strain O153 [27]. The *iif* genes of this strain are involved in the conversion of indole to anthranilate. The indole degradation process involves hydroxylation to a dihydrodiol compound by the flavin-dependent two-component oxygenase system, which includes IifC and IifD. The dihydrodiol compound is further degraded by IifB. Subsequently, anthranilate is further degraded via aromatic ring cleavage and the β-ketoadipate pathway. Based on their amino acid sequence similarities, *dcdA* and *dcdB* are thought to encode the oxygenase and reductase components, respectively, of aromatic hydroxylating dioxygenase, which is involved in 34DCA hydroxylation. Notably, the enzymatic reaction analysis of the *dcdA* and *dcdB* gene products revealed that compound I is generated from 34DCA. Therefore, compound I appears to be a diol compound produced from 34DCA via hydroxylation catalyzed by DcdAB. The disruption of the *dcdA* and *dcdB* genes confirmed their essential roles in 34DCA degradation in strain GFJ2.

The deduced amino acid sequence of *dcdC*, which is located upstream of *dcdA*, was similar to that of short-chain dehydrogenases, such as IifB from strain O153 and BacC from *Bacillus subtilis* strain 168 [27,28]. Therefore, the gene product is likely involved in dehydrogenation reactions. Moreover, compound I was converted to 4,5-dichlorocatechol upon the addition of DcdC, suggesting that DcdC acts as a dehydrogenase that oxidizes compound I to 4,5-dichlorocatechol. These results indicate that 34DCA is converted to 4,5-dichlorocatechol via diol compound (compound I) catalyzed by DcdAB and DcdC. It has been reported that catechol/chlorocatechol 1,2-dioxygenase activity is induced during the growth of strain GFJ2 on 34DCA [15]. Therefore, 4,5-dichlorocatechol generated from 34DCA is possibly further degraded via the catechol/chlorocatechol 1,2-cleavage pathway in this strain. The genome sequence analysis of strain GFJ2 revealed that the BEN76_06755 gene encodes an enzyme homologous to the putative catechol 1,2-dioxygenase. Based on this result, the BEN76_06755 gene product might be involved in the degradation of 34DCA as an aromatic ring cleavage of catechol or chlorocatechol. Unfortunately, the functions of BEN76_RS01175 and BEN76_RS01195, which are included in the *dcd* gene cluster, were not identified in this study. However, these genes may play a role in the degradation of 34DCA in this strain since they are part of the *dcd* operon. Hongsawat et al. suggested that strain GFJ2 degrades 34DCA to 4-chloroaniline via a dechlorination reaction. 4-chloroaniline is then further degraded via hydroxylation and aromatic ring cleavage reactions [15]. This proposed pathway differed from that observed in this study. The results of our gene disruption experiment and analysis of the gene product expressed in a heterologous host revealed that 34DCA is converted to 4,5-dichlorocatechol via initial hydroxylation and dehydrogenation reactions. The degradation pathway of 34DCA to acetanilide in *B. megaterium* IMT21 has been reported [13]. However, the related genes and enzymes of this pathway in strain IMT21 are currently unknown. Therefore, it was not possible to compare the differences in genetic-level 34DCA degradation between strains GFJ2 and IMT21. *P. putida* T57 can utilize 4-chloroaniline and degrades it via a hydroxylation reaction catalyzed by the dioxygenase component [12]. Therefore, it is suggested that, in this strain, 4-chloroaniline degradation may proceed similarly to the 34DCA degradation of GFJ2. Hydroxylation and ring cleavage reactions are also involved in the degradation of aniline in many bacteria [14]. It has been reported that dichloroaniline degradation is enhanced in the presence of aniline in 34DCA-utilizing *Pseudomonas putida* [29]. Therefore, a common metabolic pathway including aromatic cleavage reaction might be involved in both dichloroaniline and aniline degradation.

In the present study, transcriptional analyses indicated that the *dcd* gene cluster was constructed as an operon, induced when strain GFJ2 was grown on 34DCA, providing further evidence for their involvement in the utilization process. The downstream region of the *dcd* operon, the BEN76_01200 gene, which encodes a putative transcriptional regulator, is located in the opposite direction. The deduced amino acid sequence of this gene was similar to that of the *iifR* gene in strain O153 and *A. baumannii* strain ATCC 19606. The *iifR* gene product is an AraC family transcriptional regulator involved in the transcriptional induction of the *iif* genes [30]. AraC family proteins are known to play roles as positive transcriptional regulators in many bacteria [31]. AraC proteins from *E. coli* recognize the promoter region of the arabinose degradation gene operon (*araBAD*) to regulate its transcription. AraC is known to control the activation and repression of transcription of the *ara* operon by binding to different regions in the promoter, depending on the presence of arabinose [32,33,34]. Based on its sequence similarity, the BEN76_01200 gene is thought to code for an AraC regulator and likely regulates the transcription of *dcd* genes, which are responsible for the presence of 34DCA in strain GFJ2. The 34DCA-degrading gene cluster of *Variovorax* sp. strain SRS16 contains *dcaR*, which encodes a LysR-type transcriptional regulator [11]. However, the involvement of this regulator in the transcription of *dca* genes in strain SRS16 remains to be elucidated. It is imperative to elucidate the transcriptional regulatory mechanisms governing the *dcd* operon to better characterize the 34DCA-degrading system of strain GFJ2.

The present study provides important insights into the effective application of *A. soli* GFJ2 for the remediation of phenylurea-herbicide-contaminated environments. In the future, the identification of the transcriptional regulatory mechanisms of the *dcd* operon will provide further understanding of the 34DCA degradation system of strain GFJ2.

## Figures and Tables

**Table 1:** Characteristics of putative 34DCA degradation genes.

Gene	Deduced Molecular Mass (Da) ^a^	Representative Homolog	Identity (%) ^b^	Accession No.
BEN76_01175	45,749 (415)	Dienelactone hydrolase family protein (IifA) from *Acinetobacter* sp.	89	ARO76326
		Dienelactone hydrolase from *Azospirillum brasilense*	22	Q43914
		Dienelactone hydrolase from *Pseudomonas putida*	20	P0A114
BEN76_01180 (*dcdC*)	28,223 (263)	Short-chain dehydrogenase (IifB) from *Acinetobacter* sp.	81	ARO76327
		Dihydroanticapsin 7-dehydrogenase (BacC) from *Bacillus subtilis*	30	P39640
		3-Oxoacyl-[acyl-carrier-protein] reductase (FabG) from *Aquifex aeolicus*	30	O67610
BEN76_01185 (*dcdA*)	46,014 (412)	Indole dioxygenase (IifC) from *Acinetobacter* sp.	87	ARO76328
		Styrene monooxygenase (StyA) from *Pseudomonas* sp.	29	O50214
BEN76_01190 (*dcdB*)	19,036 (172)	Flavin reductase (IifD) from *Acinetobacter* sp.	90	ARO76329
		Reductase component (HsaB) of 3-hydroxy-9,10-secoandrosta-1,3,5(10)-triene-9,17-dione 4-hydroxylase from *Rhodococcus jostii*	31	Q0S808
		Reductase component (NtaB) of *p*-hydroxyphenylacetate 3-hydroxylase from *Acinetobacter baumannii*	22	Q6Q271
BEN76_01195	34,850 (315)	MetA-pathway of phenol degradation (IifE) from *Acinetobacter* sp.	83	ARO76330
		Hypothetical protein from *Acinetobacter baumannii*	80	ENW75085
BEN76_01200	40,833 (353)	AraC-type transcriptional regulator (IifR) from *A. baumannii*	73	F911_02001
		AraC/XylS-type transcriptional regulator (AntR) from *Burkholderia cepacian*	23	Q84BZ4

^a^ The values in parentheses are the numbers of amino acid residues. ^b^ Percentage identity calculated by aligning the deduced amino acid sequences using the EMBOSS alignment tool.

## Data Availability

The derived data supporting the findings of the current study are available from the corresponding author upon reasonable request. The genome sequence of *A. soli* strain GFJ2 was deposited in DDBJ/EMBL/GenBank under the accession numbers CP016896, CP016897, CP016898, CP016899, CP016900, CP016901, CP016902, and CP016903 for chromosomes pGFJ1, pGFJ2, pGFJ3, pGFJ4, pGFJ5, pGFJ6, and pGFJ7, respectively.

## Supplementary Materials

The following supporting information can be downloaded at: https://www.mdpi.com/article/10.3390/microorganisms12030613/s1, Figure S1: HPLC chromatogram of the authentic 4,5-dichlorocatechol.
