# Supplementary figures and images for: Characterization of the 3,4-Dichloroaniline Degradation Gene Cluster in Acinetobacter soli GFJ2
Source: Microorganisms. 2024 Mar 19;12(3):613. doi: 10.3390/microorganisms12030613 (PMC10974035; doi:10.3390/microorganisms12030613)

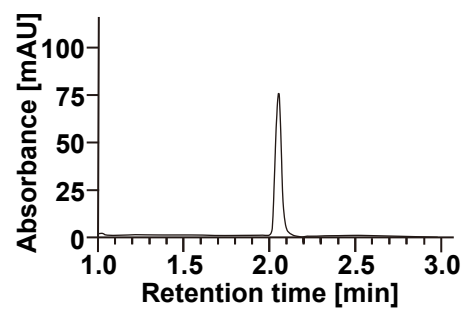

**Figure. S1**

**Figure. S1** HPLC chromatogram of the authentic 4,5-dichlorocatechol

Supplement: Supplementary file 1 [file microorganisms-12-00613-s001.zip › microorganisms-2908159-supplementary.pdf]
